# Insulin resistance and obesity among infertile women with different polycystic ovary syndrome phenotypes

**DOI:** 10.1038/s41598-017-05717-y

**Published:** 2017-07-13

**Authors:** Moamar Al-Jefout, Nedal Alnawaiseh, Aiman Al-Qtaitat

**Affiliations:** 1 Department of Obstetrics and Gynaecology, Mutah Medical Faculty, Mutah University, Mutah, Jordan; 2 Department of Obstetrics and Gynaecology, CM&HS, UAEU, Al-Ain, UAE; 3 Department of Public Health, Mutah Medical Faculty, Mutah University, Mutah, Jordan; 4 Department of Anatomy and Histology, Mutah Medical Faculty, Mutah University, Mutah, Jordan

## Abstract

Polycystic ovary syndrome (PCOS) is a common problem among Arab women and is the main cause of infertility due to anovulation. This study investigates insulin resistance (IR) and obesity in different PCOS phenotypes among infertile women (n = 213), of whom 159 had PCOS and 54 women without PCOS, recruited as a control group. Biometric, hormonal and clinical parameters were studied. IR was observed in 133 (83.6%) women with PCOS and in 25 (46.3%) women without PCOS (p < 0.001). IR was significantly associated with PCOS only among women with central obesity (χ^2^ = 35.0, p < 0.001) and not for the normal category (χ^2^ = 4.04, p < 0.058). The LH/FSH ratio was not significantly different among the PCOS group (n = 37, 23.3%) compared to the control group (n = 9, 16.7%) (p = 0.308). Among women with PCOS, the most common phenotype was type I (50.3%), with type III (29.6%), type II (14.5%) and type IV (5.7%). Type I had the highest values of fasting insulin (median = 12.98 mU/mL) and HOMA IR values (significant difference among the four phenotypes, p = 0.009 and 0.006, respectively) and is associated with severity of the disease. There was no difference in glucose levels.

## Introduction

Polycystic ovary syndrome (PCOS) is a heterogeneous condition, the pathophysiology of which appears to be a multifactorial, polygenic and multisystem endocrine disorder affecting 5–10% of women of reproductive age, characterised by hyperandrogenism and chronic anovulation^1^. The prevalence of PCOS varies with ethnicity^2^, appearing in 6.6% in the population of the south-eastern United States^3^, 6.8% in Greece^4^, 6.5% in Spain^5^, 13% among Mexican American women^6^, and 52% among South Asian female immigrants of Britain^7^.

Clinical features of PCOS include hirsutism; androgenic alopecia^8^ menstrual irregularity, usually from the time of menarche^9^; acne^10^; hyperinsulinemia^11^; insulin resistance (IR); early onset of type 2 diabetes mellitus^12^; and dyslipidemia^13^. According to the 1990 NICHD definition, women with PCOS may present three phenotypes: (i) oligo-ovulation, hyperandrogenemia and hirsutism (Oligo+ HA+ Hirsutism); (ii) oligo-ovulation and hyperandrogenemia, without frank hirsutism (Oligo+ HA); and (iii) oligo-ovulation and hirsutism, without measurable hyperandrogenemia (Oligo+ Hirsutism)^14^. According to ESHRE guidelines^15^, women with PCOS present with four phenotypes: type I: hyperandrogenism, chronic anovulation, and polycystic ovaries; type II: hyperandrogenism and chronic anovulation but with normal ovaries; type III: hyperandrogenism and polycystic ovaries but ovulatory cycles; and type IV: chronic anovulation and polycystic ovaries but no clinical or biochemical hyperandrogenism.

The association between PCOS and hyperinsulinemia was first reported by Burghen *et al*.^16^, as it became clear that women with the syndrome have major metabolic as well as reproductive morbidities. Recently, more attention was focused on the degree of IR (insulin resistance) in women with PCOS. One report even considered all women with PCOS to have some degree of IR^17^. Recent evidence suggests that obesity appears to exert an additive synergistic impact on the manifestations of PCOS, including a modifying effect on insulin sensitivity and gonadotrophin secretion and independently and negatively affecting insulin sensitivity, risk of diabetes, and cardiovascular impact^18^.

This study aimed to examine the prevalence of different PCOS phenotypes among infertile women with PCOS and to investigate the prevalence of insulin resistance and obesity in different PCOS phenotypes compared with infertile women without PCOS.

## Results

A total of 213 infertile Jordanian women with or without PCOS were studied; 159 were diagnosed with PCOS, and 54 were infertile without PCOS, who served as controls. Anthropometric characteristics of studied groups are presented in Table 1. The median age and age at menarche were 24 and 13 years, respectively, and were not significantly different between women with versus those without PCOS. No difference was observed in body mass index (BMI) and waist circumference (WC) values or categories between PCOS phenotypes. All patients who participated in this study attended the clinic exclusively because of infertility. One hundred fifty-three women (71.8%) presented with primary infertility, and 60 women (28.2%) presented with secondary infertility. One hundred fifty-four women (72.3%) had been infertile for 2–4 years; the rest (*n* = 59, 27.7%) had been infertile for more than 4 years, with no significant difference by duration of infertility (Table 1).

**Table 1:** Demographics, PCOS features & hormonal characteristics comparison in all study subjects (N = 213).

Variable	All patients (N = 213)	PCOS (n = 159)	Control (n = 54)	*P* value
Age (y), median (IQR)	24 (22–28)	24 (22–29)	24 (22.8–25.3)	0.370
Age at menarche (y), median (IQR)	13 (12–14)	13(12–14)	13 (13–14)	0.582
Height (cm), mean (SD)	163.96 (6.2)	163.5 (5.87)	165.3 (7.0)	0.067
Weight (kg), mean (SD)	74.79 (11.85)	74.73 (11.77)	74.9 (12.2)	0.911
BMI, mean (SD)	27.91 (4.75)	28 (4.51)	27.6 (5.4)	0.644
Waist circumference (cm), mean (SD)	79.5 (9.46)	80.49 (9.47)	76.6 (8.9)	0.017
Characteristic/Duration of infertility, % (n)				
>2–4 years	72.3 (154)	72.3 (115)	72.2 (39)	0.988
5 or more years	27.7 (59)	27.7 (44)	27.8 (15)	
Androgenic alopecia	Yes	59 (37.1%)	3 (5.6%)	<0.001
Acanthosis Negricans (AN)	Yes	45 (28.3%)	9 (16.7%)	0.089
mFG score	>8	85 (53.5%)	0 (0.0 %)	<0.001
	<8	74(46.5%)	54 (100%)	
Clinical Hyperandrogenism	None	65 (40.9%)	52 (96.3%)	<0.001
	Hirsutism and/or Acne	51(32.1%)	0 (0.0%)	
	Hirsutism	34 (21.4%)	0 (0.0%)	
	Acne	9 (5.7%)	2 (3.7%)	
Menstrual cycle regularity	Regular	52 (32.7%)	51(94.4%)	<0.001
	Oligo/amenorrhea	107 (67.3%)	3 (5.6%)	
Ovarian volume in mL	>10 mm^3^	124 (78.0%)	7 (13.0%)	<0.001
	<10 mm^3^	35 (22.0%)	47 (87.0%)	
Insulin Resistance -IR	<2.5	26 (16.4%)	29 (53.7%)	<0.001
	>2.5	133 (83.6%)	25 (46.3%)	
LH/FSH Ratio	≤2	122 (76.7%)	45 (83.3%)	0.308
	>2	37 (23.3%)	9 (16.7%)	
		**PCOS (n=159)**	**Control (n=54)**	**t-test &** ***P*** **value**
FSH(units per L) Mean		5.97	5.66	1.425^*^
				0.156**
COMPUTE FAI=TT/SHBG Mean		5.35	3.93	5.982
				0.000^**^
Fasting Insulin (microunits per mL)		1733.5***		<0.001^****^
Fasting Glucose (mg/dL)		3850.5***		0.256^****^
HOMA IR		1875***		<0.001^****^
Free Testosterone (ng/dL)		2132***		<0.001^****^
Total Testosterone (ng/dL)		1524.5***		<0.001^****^
SHBG (nanomoles per L)		4020***		0.484^****^
LH (units per L)		3102.5***		<0.001^****^

*Parametric test (normal distribution) -**Independent sample Mann-Whitney U t-test ***Grouping Variable: Cases vs. Control, ****Nonparametric test- Mann-Whitney U test.

### Prevalence of different PCOS phenotypes

The most common phenotype in our study was type I (full-blown PCOS) 50.3% (*n* = 80/159), followed by type III (women with hyperandrogenism, polycystic ovaries and normal ovulatory cycles; H/PCO-ovulatory PCOS) 29.6% (*n* = 47/159). Twenty-three women had chronic anovulation and hyperandrogenism but normal ovarian morphology, type II (H/O), 14.5% (*n* = 23/159) and, finally, only 5.7% (*n* = 9/159) of patients had type IV (O/PCO, no hyperandrogenism but chronic anovulation and polycystic ovaries). Type III and IV phenotypes represent the newer phenotypes according to the Rotterdam criteria.

### Obesity and insulin resistance among study participants

Women were divided based on the BMI and waist circumference categories as normal, overweight or obese. The mean BMI value for all participants was 27.91 (SD = 4.75); for women with PCOS, BMI was 28 (SD = 4.51) and for controls 27.6 (SD = 5.4) with no statistically significant difference (P = 0.644). However, waist circumference mean values were for all women 79.5 cm (SD = 9.46), women with PCOS 80.49 cm (SD = 9.47), controls 76.6 cm (SD = 8.9), with a *p*-value of 0.017 (Table 1). PCOS and control groups were comparable in terms of BMI; in the three categories (normal, overweight, obese), there were no significant differences between cases and controls (*p*-value = 0.822). However, there were significant differences in the waist-circumference categories (normal, overweight, obese); mainly, the obese status *n* = 40 (25.2%) compared to the obese of the control group *n* = 4 (7.4%), *p*-value = 0.018 (Table 2).

**Table 2:** Weight categories among PCOS vs. control group.

		Cases Vs Control		
		PCOS n (%)	Control n (%)	p-value*
BMI- category	Normal	49 (30.8%)	17 (31.5%)	0.822
	Overweight	60 (37.7%)	18 (33.3%)	
	Obese	50 (31.4%)	19 (35.2%)	
Waist Circumference	Normal	67 (42.1%)	26 (48.1%)	0.018
	Overweight	52 (32.7%)	24 (44.4%)	
	Obese	40 (25.2%)	4 (7.4%)	

*chi-square test (χ^2^).

As expected, androgenic alopecia *n* = 59 (37.1%) was more common among the PCOS group compared to the control group *n* = 3 (5.6%) (*p*-value < 0.001). Acanthosis nigricans was not statistically significant (*p*-value = 0.089). The modified Ferriman-Gallwey (mFG) score > 8, *n* = 85 (53.5%) was significantly higher in the PCOS group compared to the control group (*p*-value < 0.001). Hirsutism and/or acne were the highest among all hyperandrogenism categories (none, hirsutism and/or acne, hirsutism, acne) and were significantly associated with hyperandrogenism in comparison to the control group (*p*-value < 0.001). The total number of women with oligomenorrhea or amenorrhea was 107 (67.3%) and was significantly higher in women with PCOS compared to the control group (*p*-value < 0.001). Ovarian volume of more than 10 mm^3^ was *n* = 124 (78.0%) in the PCOS group, which was significant compared to the control group (*n* = 7, 13.0%) with *p*-value < 0.001. Insulin resistance (IR > 2.5) (*n* = 133, 83.6%) was also more common among the cases of PCOS compared to the control group (*p*-value < 0.001). However, when we compared the luteinising hormone/ follicle-stimulating hormone (LH/FSH) ratio (cut-off > 2), we found that it was higher among the PCOS group (*n* = 37, 23.3%) than in the control group (*n* = 9, 16.7%), but this difference was not statistically significant (*p*-value = 0.308) (Table 1).

Women with PCOS have higher median rank fasting insulin, HOMA-IR, free testosterone and total testosterone than controls (*p*-value < 0.001). However, fasting glucose, sex hormone binding globulin (SHBG) and FSH values were not statistically significant (Table 1). Free androgen index (FAI) was significantly higher in the PCOS group (5.35 vs. 3.93, *p*-value = <0.001) (Table 1).

We explored BMI and waist circumference among all study participants and found that both were significantly higher among those with IR of >2.5 compared to those with IR of <2.5. It was particularly clear among the obese, (*n* = 63, 39.9%) and (*n* = 41, 25.9%) for BMI and waist circumference, respectively (*p*-value < 0.001). Obesity was associated with IR in all cases and controls (Table 3). Cochran–Mantel–Haenszel statistics was chosen to assess the significance of association between the disease status (cases vs. control) and IR (>2.5 vs. <2.5) while controlling for BMI or waist circumference status (normal vs. obese) (Table 4). The results of a stratified χ^2^ test with test of conditional independence (Cochran–Mantel–Haenszel) for IR (>2.5 vs. <2.5) and the clinical status (cases vs. controls) while controlling for BMI status (normal vs. obese) showed that IR > 2.5 was significantly associated with PCOS for normal BMI (χ^2^ = 6.60, *p* = 0.022), whereas for the obese category, it was also significant (χ^2^ = 27.12, p < 0.001). Mantel-Haenszel statistics were significant with χ^2^ = 29.08 and p < 0.001. IR > 2.5 was significantly associated with the PCOS only among the obese category of waist circumference (χ^2^ = 35.0, *p* < 0.001). For the normal category, it was not significant (χ^2^ = 4.04, *p* < 0.058). Mantel-Haenszel statistics were significant with χ^2^ = 26.48 and *p* < 0.001 (Table 4).

**Table 3:** Comparison of study subjects BMI and Waist circumference according to insulin resistance (IR <2.5 vs. >2.5).

Variable	Category	Insulin Resistance -IR		p-value
		<2.5	>2.5	
BMI- category	Normal	29 (52.7%)	37 (23.4%)	<0.001^*^
	Overweight	20 (36.4%)	58 (36.7%)	
	Obese	6 (10.9%)	63 (39.9%)	
Waist Circumference	Normal	35 (63.6%)	58 (36.7%)	<0.001^*^
	Overweight	17 (30.9%)	59 (37.3%)	
	Obese	3 (5.5%)	41 (25.9%)	

*Chi-square test.

**Table 4:** Stratified χ^2^ (Mantel–Haenszel): IR *Cases vs. Control according to BMI & Waist circumference.

					Cases Vs Control				Tests of Conditional Independence
					PCOS	Control	χ^2^	p-value	Mantel-Haenszel χ^2^ (p-value)
BMI	Normal	IR	<2.5	Count	17	12	6.60	0.022	29.084 (<0.001)
				%	34.7%	70.6%			
			>2.5	Count	32	5			
				%	65.3%	29.4%			
	Obese	IR	<2.5	Count	9	17	27.1	<0.001	
				%	8.2%	45.9%			
			>2.5	Count	101	20			
				%	91.8%	54.1%			
Waist circumference	Normal	IR	<2.5	Count	21	14	4.04	0.058	26.48 (<0.001)
				%	31.3%	53.8%			
			>2.5	Count	46	12			
				%	68.7%	46.2%			
	Obese	IR	<2.5	Count	5	15	35.0	<0.001	
				%	5.4%	53.6%			
			>2.5	Count	87	13			
				%	94.6%	46.4%			

These findings support the role of IR as a causative mechanism for the presence of PCOS in addition to the waist circumference as a predictor of the presence of PCOS. These results also confirm that it has a greater predictability than BMI. However, the additive effect of obesity in general could not be excluded, given significant variations in IR among cases compared to controls.

### Prevalence of different PCOS phenotypes

#### Hormonal, metabolic and ovarian abnormalities among different PCOS phenotypes

Among women with PCOS, we found that the median of the fasting insulin values was 12.98 mU/mL for all phenotypes, with highest values among those women with type I, and there was a statistically significant difference between phenotypes (*p*-value = 0.009). Fasting glucose did not differ between the groups. HOMA-IR, FSH, LH and FSH/LH ratio were statistically different between the groups. Phenotype IV had the highest LH and LH/FSH ratio, whereas type I had the highest HOMA-IR values. When we compared FSH levels, using post hoc Bonferroni comparisons between different phenotypes, there were statistically significant differences in FSH levels between phenotypes I and II and between I and III (*p* < 0.01) (Table 5).

**Table 5:** Main hormonal profiles for patients with PCOS (N = 159).

Hormonal profile	All PCOS patients (N = 159)	Type I (n = 80)	Type II (n = 23)	Type III (n = 47)	Type IV (n = 9)	*P* value
Fasting insulin (microunits/mL)	12.98 (11.87–15.79)	13.86 (12.73–16.86)	12.00 (10.65–14.75)	12.70 (10.80–13.97)	12.90 (11.83–13.87)	0.009
Fasting glucose (mg/dL)	98.0 (89.0–100.0)	98.0 (89.0–100.0)	97.0 (89.0–100.0)	97.0 (89.0–100.0)	97.0 (87.0–99.5)	0.819
HOMA-IR	3.11 (2.72–3.64)	3.38 (2.92–3.96)	2.86 (2.53–3.51)	2.95 (2.45–3.38)	2.85 (2.60–3.48)	0.006
Free testosterone (ng/dL)	0.87 (0.75–0.96)	0.87 (0.75–0.96)	0.86 (0.54–0.87)	0.87 (0.75–0.97)	0.88 (0.87–1.87)	0.052
Total testosterone (ng/dL)	87.90 (66.90–99.44)	87.87 (66.90–99.33)	80.98 (67.90–98.09)	87.90 (66.90–100.87)	87.90 (79.04–117.80)	0.522
SHBG (nanomoles/L)	56.76 (54.32–65.87)	55.60 (54.32–65.87)	56.76 (54.32–63.98)	56.87 (54.76–65.76)	55.95 (44.76–65.82)	0.377
FAI, mean (SD)	5.35 (1.48)	5.37 (1.50)	5.17 (1.37)	5.07 (1.24)	6.29 (2.38)	0.134
LH (units/L)	9.87 (8.76–11.75)	10.86 (9.01–11.76)	9.76 (8.65–10.87)	8.76 (7.65–10.87)	10.99 (9.81–12.65)	0.001
FSH (units/L), mean (SD)	5.97 (1.44)	6.47 (1.51)	5.41 (1.17)	5.43 (1.20)	5.65 (1.03)	<0.001
LH/FSH ratio	1.66 (1.50–1.98)	1.58 (1.40–1.86)	1.89 (1.50–2.33)	1.65 (1.50–1.97)	2.05 (1.67–2.23)	0.017

N.B. all summary data are presented as median and inter-quartile range except FAI and FSH (mean and standard deviation).

When examining the values of HOMA-IR in all PCOS patients, we found that HOMA-IR values differ significantly, with high BMI and WC values with *p*-value < 0.001 (Table 3).

As expected, women with type I had the highest prevalence of PCO and large ovarian volume, and there was a statistically significant difference between the three phenotypes (type II excluded because there should not be features of PCO on U/S scan) with *p* < 0.001.

#### PCOS patient distribution according to BMI and waist circumference categories

The distribution of patients among weight categories shows women with PCOS with waist circumference categories Normal (<79.9 cm) = 67 women (42.1%), overweight (80.0 to 87.9 cm) = 53 women (33.3%) and obese (>88 cm) = 39 women (24.5%), whereas according to WHO BMI categories, Normal (<25 Kg/m^2^) = 48 women (30.2), overweight (>25 < 30 Kg/m^2^) = 61 women (38.4%) and obese (>30 Kg/m^2^) = 50 women (31.4%). When exploring the percentages of BMI and WC categories in relation to HOMA-IR (>2.5) (Table 3), the percentage of women with HOMA-IR > 2.5 and normal weight was 23.3% in the BMI category and 34.6% in the WC category. One-third of overweight women in both BMI and WC categories had IR, whereas all obese women in both categories had HOMA-IR > 2.5, indicating that they were insulin-resistant. Both BMI and WC were concordant using a correlation coefficient between groups regarding HOMA-IR with *p* < 0.001 (BMI and WC rs = 0.40 and 0.39, respectively). Moreover, Table 2 shows subgroups of PCOS patients divided based on the BMI, normal, overweight, and obese PCOS. The percentage of normal BMI category (BMI < 25 kg/m2) was 30.2% (48/159), overweight women (BMI ≥ 25 and <30) was 38.4% (61/159) and obese women (BMI > 25 kg/m2) was 31.4% (50/159).

#### Insulin resistance (IR) among different PCOS phenotypes

There was even distribution of HOMA-IR values between obese and non-obese women with PCOS, using independent Samples Kruskal-Wallis test of the distribution of HOMA-IR with *p*-value < 0.005 (Table 5). In addition, HOMA-IR did significantly differ among the four phenotype groups, using independent Samples Kruskal-Wallis test of the distribution of HOMA-IR with *p*-value = 0.006 (Table 5).

#### Clinical hyperandrogenism associated with BMI in PCOS patients

Concerning clinical hyperandrogenism features, our results showed that androgenic alopecia was associated with only obesity among all PCOS subjects: normal weight 0% (*n* = 0), overweight: 41.0% (*n* = 25), obese: 68.0% (*n* = 34) with *p*-value < 0.001.

## Discussion

Our study shows that the type I classical and full-blown severe PCOS phenotype is the most common; this accords with other studies. In addition, the prevalence of all phenotypes is similar to the findings of other studies^19, 20^. Our results show that fasting insulin, fasting glucose and free testosterone were statistically different between weight categories in PCOS patients. Women with the type I PCOS phenotype were also found to have more insulin resistance and higher values of free testosterone, LH, FSH and LH/FSH ratio. On the other hand, our Jordanian study is similar in prevalence of type I PCOS phenotype to an Italian study^21^, in contrast to Iranian^22^ and Chinese^23^ studies, in which the type IV PCOS phenotype was the most prevalent (Table 6).

**Table 6:** Comparison of PCOS phenotypes in different ethnic groups.

	Type I H + O + PCO	Type II H + O	Type III H + PCOS	Type IV O + PCO
In our study (%)	50%	14.6%	28.9%	5.7%
Chinese study (%)^23^	26.8%	7.6%	13.4%	52.2%
Italian study (%)^21^	53.9%	8.9%	28.8%	8.4%
Iranian study (%)^22^	32.1%	14.8%	4.3%	46.8%

As demonstrated in our data, obesity is more prevalent in women with the classic severe PCOS phenotype. Thus, women with PCOS Type I; are at increased risk of developing insulin resistance features, which is independent and may be worsened by central adiposity. In addition, obese women have higher free testosterone levels than women with normal weight. Our data also shows that waist circumference measurement is a better predictor of central obesity than BMI. Several studies have described endocrine and metabolic differences between lean and obese women with PCOS. In addition to alteration in insulin sensitivity that was independent of obesity, these studies have demonstrated more marked hyperandrogenemia, IR and relative hyperglycemia and lower sex hormone binding globulin (SHBG) in the obese compared with lean women with PCOS^24^.

Obesity is a common problem among Jordanian females^25^. Although polycystic ovary syndrome is believed to be one of the most common endocrine disorders in women worldwide, reports are very rare regarding the clinical and biochemical features of Arabic (including Jordanian) women with PCOS^26^. The hormonal profile of PCOS among Saudi women was similar to that published in the literature, with the exception of the prevalence of metabolic syndrome, which was less than in global reports^27^. As shown above, HOMA-IR was significantly correlated with BMI. In addition, our advanced statistical analysis showed that IR is highly associated with the presence of PCOS, although the additive synergistic effect of obesity could not be excluded. The results also show that obesity would worsen the severity of insulin resistance. The prevalence of insulin resistance was not significantly different between various PCOS phenotypes and type I, the most severe phenotype had the highest values of IR, in agreement with other reports^28^. Obese women with PCOS have more severe hyperandrogenism and its related clinical features (such as hirsutism, menstrual abnormalities and anovulation) than normal-weight women with PCOS. This picture tends to be more profound^29^ particularly in obese women with PCOS and central obesity.

Many patients with PCOS demonstrate other metabolic abnormalities. Especially notable is the presence of insulin resistance (IR), accompanied by compensatory hyperinsulinemia^30–33^. Actually, insulin possesses true gonadotrophic function and an increase in insulin availability within ovarian tissue may enhance excess androgen synthesis. Obesity, particularly the abdominal type, may be partly responsible for insulin resistance and associated hyperinsulinemia in women with PCOS. Therefore, obesity-related hyperinsulinemia may play a key role in favouring hyperandrogenism^34^. Moreover, obesity is associated with IR^35^. IR is also affected by ethnicity^36^ and age^37^. When we used the HOMA-IR calculation to estimate insulin sensitivity, adjusted for confounders, we observed that 133 (83.6%) of 159 PCOS patients were insulin-resistant. This is consistent with the recent findings of other reports^38, 39^. Furthermore, insulin resistance is present in both obese and non-obese women with PCOS^40^. Legro *et al*.^41^ reported a higher prevalence of insulin resistance in obese (64%) than in non-obese (20%) women with PCOS. In our study, the prevalence of insulin resistance was significantly higher in overweight patients than in patients of normal weight.

As shown above, there was no difference in FSH levels between cases and controls and, in fact, there was a relative deficit in FSH levels in women with PCOS. Some reports indicate that altered secretion of inhibin B may lead to a relative deficit of FSH in PCOS^42^. The overproduction of LH and, consequently, an incorrect LH/FSH ratio is presently not considered to be a characteristic attributed to all PCOS patients. In our study, we found that LH/FSH changes were attribute of 23.3% of cases with PCOS and in 16.7% of controls, which differs from the findings of other studies, in which higher incidence was reported^29^. Moreover, our results show that obesity was not a risk factor for an abnormal LH/FSH ratio, disproving the traditional concept of the disease that the heavier the patient is, the higher the LH/FSH ratio, and our results agree with a recent report^43^.

This study has a number of strengths, including the relatively good number of patients and the richness of data, which paves the way for more studies to address this problem. In addition, our study population is homogeneous in racial and ethnic variations, which gives more credibility to the results and conclusions. Furthermore, this study included subjects with infertility with or without PCOS, in whom, unfortunately, the main focus of doctors and patients was on infertility problems; our data will encourage doctors and patients to be aware of the high prevalence and role of obesity and insulin resistance first and, second, to correct the endocrine dysfunction in those women with PCOS by reducing their weight and improving their insulin levels. The use of mFG score for the assessment of hirsutism may be considered a limitation in the study because it is a subjective issue. The other probable limitation is the use of the HOMA-IR threshold value for diagnosis of IR resistance, which may have affected the true prevalence of IR among our study subjects. However, the gold standard for establishing IR is the euglycemic hyperinsulinemic clamp, which is not suitable for large-scale clinical use; therefore, we used the HOMA-IR calculation, which correlates with the euglycemic hyperinsulinemic clamp and is often used in other reports^22^ as a marker for IR.

The full-blown phenotype of PCOS was the most common phenotype in this cohort of Jordanian women with PCOS. The prevalence of hyperandrogenism (including hirsutism, acne and biochemical hyperandrogenemia), obesity and insulin resistance were higher in this cohort than in women from other ethnicities. This fact will encourage doctors to focus on resolving obesity and IR problems in addition to infertility problems. A focus on central adiposity evaluation by waist circumference measurement instead of BMI is more valid. LH/FSH ratio should not be part of PCOS assessment. Owing to Arabic women, including Jordanians, being at high risk developing abnormal glucose metabolism and type II diabetes, further research is required to investigate the value of the recommendation for initial and periodic screening for hyperglycemia and IR in women with PCOS, including annual evaluation for diabetes.

Incorporating information from our study, we can now characterise the phenotype of Arabic women with PCOS as being similar to other women with PCOS with regard to insulin resistance and obesity, but the key differences are that our women have more of types I and III and less of type IV phenotypes.

## Methodology

### Subjects and setting

This cross sectional observational study was carried out in Karak City in the southern part of Jordan from January 2012 to April 2015. A total of 219 consecutive, untreated women presenting with initial diagnosis and treatment of infertility due to different causes, including PCOS, were recruited. The diagnosis of PCOS was determined according to the Rotterdam’s criteria^15^. Patients’ median age was 24 (IQR: 22–29) years. Due to infertility problems, none had used oral contraceptives for at least 3 months preceding the study. Women with persistent elevations of prolactin (PRL) (>24 μg/L) or abnormal thyrotropin (TSH) values (>5.5 mIU/L or <0.35 mIU/L) were excluded. Six cases were excluded, leaving a total final number of 213 study subjects. Of them, 159 had infertility problems due to PCOS; 54 women had infertility problems due to other reasons, including tubal factor, endometriosis and pelvic inflammatory disease, and these women served as a control group. The causes of infertility were determined by clinical evaluation, laboratory investigations, hysterosalpingogram and hysteroscopy or laparoscopy.

This study was based in two gynaecological clinics, one at the Ministry of Health in Al-Karak Hospital, affiliated with the Mutah University Obstetrics & Gynaecology Department in the south of Jordan, and the other at the main author’s (MA) private practice. Institutional review board approval was obtained from Mutah University Ethics Committee (N0-201217), and informed written consent was obtained from all study participants. We confirm that all methods were performed in accordance with the relevant guidelines and regulations of the ethics in Human research and all research data can be available.

### Protocol

A standardised form was used to take medical history and physical examination, with emphasis on menstrual dating and regularity, the presence of hirsutism, acne and acanthosis nigricans. Full records of medications and gynaecological and family histories were obtained. Menstrual cycles were identified as (1) regular menses: a cycle with an inter-menstrual interval of 21 to 35 days; (2) oligomenorrhea: an inter-menstrual interval of 36 days or longer; and (3) amenorrhea: an inter-menstrual interval of 6 months or longer in previously regularly menstruating women and more than 3 months in women with oligomenorrhea.

Women diagnosed with infertility were grouped into two groups: those with PCOS and those without PCOS. The diagnosis of PCOS was based on clinical, laboratory and ultrasound scan data (see ovarian assessment). All women underwent physical examination. Obesity was assessed by estimating body mass index (BMI: weight/height^2^ in kg/m^2^) with normal range between 17 and 25, overweight between 25 and 30 and obese >30. Central obesity was assessed by waist circumference (WC) measurements according to the WHO protocol^44^, measured at the mid-point between the highest point of the iliac crest and the last palpable rib, using a flexible, inelastic measuring tape with a tension meter attached. Then all measures were classified into three categories: normal (WC 79.9 cm or less), overweight (WC 80.0 to 87.9 cm) and obese (WC 88.0 cm or more), according to age- and sex-specific cut-offs^45^.

On physical examination, the presence of terminal hair growth was scored using a modification of the Ferriman-Gallwey (mFG) method with a score of 8 or greater (mFG ≥ 8) was considered hirsutism^46, 47^. The mFG scoring method represents the sum of the hair growth scores of nine body areas (upper lip, chin, chest, upper abdomen, lower abdomen, upper arms, thighs, upper back, and lower back), with the examiner assessing terminal hair growth in each area and rating it from 0 (no hair) to 4 (extensive hair growth)^48^. Biochemical hyperandrogenism was identified as a total T level >83.6 ng/dL (SI Unit: 2.96 nmol/L), free T level >0.81 ng/dL (0.026 nmol/L), free androgen index FAI = [total testosterone/sex hormone binding globulin (SHBG) × 100] > 5^49^ or a DHEAS level >1,580 ng/mL (6.64 mol/L)^50^. We defined clinical hyperandrogenism (HA) as the presence of hirsutism and/or acne; because there is no agreement regarding the best way to assess acne^51^, we elected to record its presence without scores in our study subjects.

### PCOS Phenotypes

PCOS is heterogeneous endocrine disease that can present by oligo- or anovulation (O) (progesterone <5.0 ng/mL (1 ng/mL = 3.2 nmol/L) on at least one luteal phase sample day^52^), biochemical or clinical hyperandrogenism (HA) and polycystic ovaries (PCO). Participants with PCOS were grouped according to their PCOS features in four possible groups^53^ as follows:

Type I: hyperandrogenism, chronic anovulation and polycystic ovaries (O + HA + PCO).

Type II: hyperandrogenism and chronic anovulation (O + HA).

Type III: hyperandrogenism and polycystic ovaries (HA + PCO).

Type IV: chronic anovulation and polycystic ovaries (O + PCO).

### Hormonal assessment

Blood samples were collected from all participants after having been instructed not to eat, drink or smoke. All patients reported no consumption of alcohol ever. A 10-cc sample of blood was drawn during days 2–6 of the menstrual cycles (natural or bleeding after progestin withdrawal) in plain-top tubes for subsequent hormonal analysis. If amenorrhoea was present, this was performed after excluding a dominant follicle with ultrasound. Blood was analysed for free and total testosterone (FT and TT) (FT; nmol/L), sex hormone-binding globulin (SHBG; normal values 40–120 nmol/L), LH (units per L), FSH (units per L) and fasting blood glucose (mg/dL); fasting insulin (micro-units per mL) was detected by the glucose oxidase method (AU640 automation biochemistry analyser and its relevant reagent, Olympus Company, Hamburg, Germany), sex hormone binding globulin (SHBG), luteinising hormone (LH) and follicle-stimulating hormone (FSH). LH/FSH ratio was calculated for each subject; LH/FSH ratio >2 was considered abnormal. The LH and FSH were measured by electrochemiluminescence technique in a COBAS AutoAnalyzer using reagents manufactured by Roche Diagnostics; quality control was done by PreciControl 1 and 2, and then the mean LH/FSH ratio was obtained. Free androgen index (FAI) was calculated by [total testosterone] X 100 divided by [SHBG]. Normal insulin sensitivity was defined by fasting insulin levels <12 mU/mL. We also calculated indices of the homeostasis model assessment (HOMA-IR)^54^. HOMA−IR was calculated using the equation HOMA−IR = Fasting insulin (μU/mL) × fasting glucose (mg/dL)/405^55^ with HOMA-IR > 2.5 mol μU/mL as cut-off of abnormal value^56^.

In cases of fasting glucose consistent with impaired glucose tolerance (fasting glucose [FG] 110–125 mg/dL), a 75-g oral glucose tolerance test was performed; subjects with overt diabetes mellitus and impaired glucose tolerance were referred to a diabetes specialist. In addition, serum PRL (nomograms per mL), TSH (micro-units per mL) and 17α-hydroxyprogesterone (17-αOHP; nomograms per mL) levels were examined in blood samples of women with oligomenorrhea. Normal values of 20–100 ng/dL (all blood samples were taken before ovulation on day 2 of cycle) were accepted to exclude other causes of menstrual disorders^57^. In addition, blood samples of dehydroepiandrosterone sulfate (DHEAS) ug/dL and 17-hydroxyprogesterone (OHP) were also obtained with, normal values = 380 ug/dL. If values of DHEAS were <380 ug/dL, patients with apparent adrenal problems causing hyperandrogenism were excluded.

### Ovarian assessment

The revised Rotterdam criteria were used for the diagnosis of PCO on ultrasound^58^, i.e., either an ovary with ≥12 follicles measuring 2–9 mm in diameter or an ovary with increased volume (>10 cm^3^ without concomitant cysts). Ultrasound examination was performed by one investigator (MA) during the first 5 days of the menstrual cycle (the early follicular phase). The ultrasound assessments were undertaken trans-vaginally using a computed sonography system with a 7.0-MHz transducer. After identification of the ovaries, the size of the ovary was measured in three orthogonal planes. Ovarian volume was calculated using the formula for a prolate ellipsoid. The total number of follicles in each ovary was counted. Measurements of the largest and smallest follicles were taken in their maximum diameters and recorded in mm. Follicles were counted on the frozen images of two non-overlapping planes in the longitudinal section of each ovary.

### Statistical analysis

Statistical analysis was performed using the Statistical Package for the Social Sciences (SPSS), version 20.0, for Windows (SPSS, Inc., Chicago). Data are presented as mean ± SD. When variables were significantly skewed, median and interquartile ranges were used. Categorical variables were analysed using Chi-square tests, normally distributed continuous variables using analysis of variance (ANOVA) and significantly skewed continuous variables were analysed using the Kruskal-Wallis test. Student’s *t*-test was used for the comparison of continuous variables. Group means were compared using analysis of variance (ANOVA) with post hoc least squares means pair-wise comparisons (after log transformation of the values). Statistical significance was considered when two-tailed *P* was ≤ 0.05.
